# Density of surface charge is a more predictive factor of the toxicity of cationic carbon nanoparticles than zeta potential

**DOI:** 10.1186/s12951-020-00747-7

**Published:** 2021-01-06

**Authors:** Maud Weiss, Jiahui Fan, Mickaël Claudel, Thomas Sonntag, Pascal Didier, Carole Ronzani, Luc Lebeau, Françoise Pons

**Affiliations:** 1 Laboratoire de Conception et Application de Molécules Bioactives, Faculté de Pharmacie, UMR 7199, CNRS-Université de Strasbourg, Illkirch, France; 2 Laboratoire de Bioimagerie et Pathologies, Faculté de Pharmacie, UMR 7021, CNRS-Université de Strasbourg, Illkirch, France; 3 Faculté de Pharmacie, UMR 7199, 74 route du Rhin, 67400 Illkirch, France

**Keywords:** Nanoparticle, Carbon dots, Toxicity, Charge, Surface chemistry, Lung

## Abstract

**Background:**

A positive surface charge has been largely associated with nanoparticle (NP) toxicity. However, by screening a carbon NP library in macrophages, we found that a cationic charge does not systematically translate into toxicity. To get deeper insight into this, we carried out a comprehensive study on 5 cationic carbon NPs (NP2 to NP6) exhibiting a similar zeta (ζ) potential value (from + 20.6 to + 26.9 mV) but displaying an increasing surface charge density (electrokinetic charge, Q_ek_ from 0.23 to 4.39 µmol/g). An anionic and non-cytotoxic NP (NP1, ζ-potential = − 38.5 mV) was used as control.

**Results:**

The 5 cationic NPs induced high (NP6 and NP5, Q_ek_ of 2.95 and 4.39 µmol/g, respectively), little (NP3 and NP4, Q_ek_ of 0.78 and 1.35 µmol/g, respectively) or no (NP2, Q_ek_ of 0.23 µmol/g) viability loss in THP-1-derived macrophages exposed for 24 h to escalating NP dose (3 to 200 µg/mL). A similar toxicity trend was observed in airway epithelial cells (A549 and Calu-3), with less viability loss than in THP-1 cells. NP3, NP5 and NP6 were taken up by THP-1 cells at 4 h, whereas NP1, NP2 and NP4 were not. Among the 6 NPs, only NP5 and NP6 with the highest surface charge density induced significant oxidative stress, IL-8 release, mitochondrial dysfunction and loss in lysosomal integrity in THP-1 cells. As well, in mice, NP5 and NP6 only induced airway inflammation. NP5 also increased allergen-induced immune response, airway inflammation and mucus production.

**Conclusions:**

Thus, this study clearly reveals that the surface charge density of a cationic carbon NP rather than the absolute value of its ζ-potential is a relevant descriptor of its in vitro and in vivo toxicity.
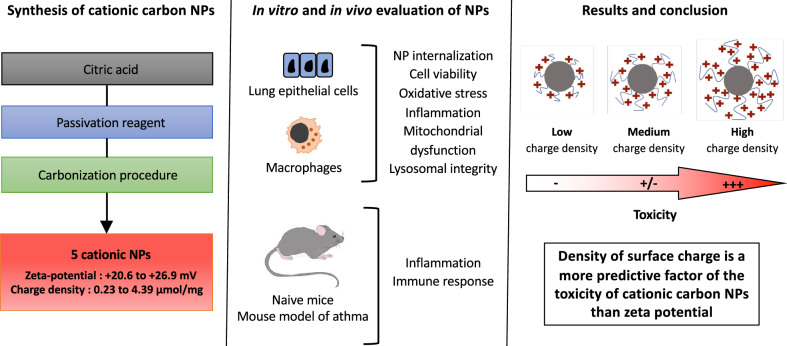

## Background

A large variety of nanoparticles (NPs), defined as particles with a size of less than 100 nm in one dimension, are currently developed for a wide range of applications including consumer goods manufacturing, foodstuff elaboration as well as medical applications [1]. These NPs are increasingly present in our professional and domestic environment, which raises the question of their potential adverse effects on human health [2]. Nanotoxicology, the branch of science that studies the hazard posed by NPs to humans and their environment aims at addressing this issue [3, 4]. However, this task is made complex by the huge diversity of NPs in terms of physicochemical characteristics, including chemical composition, size, shape, and surface charge and chemistry [1, 4]. One of the challenges of nanotoxicology is therefore to define the chemical and physical properties that drive NP adverse health effects in order to better predict NP toxicity and allow the production of safe-by-design NPs.

In the last decade, numerous studies have been conducted on the toxicological effects of engineered NPs in the lung, since inhalation is likely the main route of exposure to NPs. These studies have shown that NP inhalation may be harmful to the lung. Indeed, NPs have been reported to be cytotoxic towards lung cells such as macrophages and airway epithelial cells [5, 6]. As well, NP administration into the lung of laboratory animals was shown to induce airway inflammation, characterized by a cellular infiltrate composed of neutrophils and macrophages, and the production of pro-inflammatory cytokines [7–12]. Also, NP inhalation has been found to promote asthma onset and exacerbation, resulting in increased allergen sensitization and worsening of airway inflammation and remodeling in mouse models of the disease [13–19]. The toxicological effects of engineered NPs in the lung are however dictated by their physicochemical characteristics, such as their size and chemical composition, but also their charge, surface chemistry and shape [20, 21]. Indeed, these characteristics determine the surface reactivity of NPs, i.e. their capacity to react with their immediate environment [22, 23]. They thus influence their interaction with the components of biological media (e.g. proteins or cell surfaces), to the point of drastically modifying their fate in the organism, and their toxicity.

Among NP physicochemical characteristics, the surface charge is one of the key factors for toxicity [24]. In vitro or in vivo studies conducted on NPs of different composition (silicon-, silver-, polystyrene-, or carbon-based NPs), have shown that a positive zeta potential (ζ-potential) is associated with greater NP toxicity compared to a negative one [25–29]. This is generally attributed to a greater capacity of positively charged NPs to interact with the cell membrane through attractive electrostatic interactions with negatively charged phospholipids or membrane proteins, and to a subsequent higher NP cell uptake [4, 26, 30]. It should be noted, though, that some authors have reported lower cell uptake of cationic NPs as compared to anionic ones, which was tentatively explained by the formation of large NP agglomerates at the membrane that could not be internalized by the cell [31, 32]. By screening a library of 35 carbon-based NPs with various surface functionalizations and ζ-potential values in human macrophages, we recently demonstrated that a cationic charge is not sufficient to confer toxicity to NPs [33]. Indeed, although a significant positive correlation was found between NP toxicity and ζ-potential value, several cationic NPs exhibited no or weak toxicity, while having a marked positive ζ-potential. Some of these non toxic or slightly toxic cationic NPs displayed PEG decoration, which could explain their relative safety [34], but not all of them. Noteworthy, the cationic NPs in this library were produced by pyrolysis of various organic materials, in the presence of nitrogen-containing passivation reagents with an increasing number of amino groups, though this did not strictly translate into increasing ζ-potential values. This led us to hypothesize that the amount of positive charges on NPs (surface charge density), rather than the ζ-potential value is predictive of the NP toxicity, when these two concepts are often mixed up.

Therefore, in the present study, to get deep insight into the role of the surface charge in the toxicity of cationic NPs, we synthesized 5 cationic carbon-based NPs displaying various amounts of amino groups at their periphery. This was achieved through the pyrolysis of a mixture of citric acid and various passivation reagents, namely high molecular weight branched poly(ethylenimine) (MW = 25 kDa, bPEI25k), low molecular weight branched poly(ethylenimine) (MW = 600 Da, bPEI600), pentaethylene hexamine (PEHA), *N,N*-dimethylethylene diamine (DMEDA) and DMEDA/poly(ethylene glycol) (MW = 550 Da, PEG550). A negatively charged and non-cytotoxic NP prepared from ammonium citrate was also produced as a control. These spherical carbon nanomaterials called carbon dots (CDs) were chosen to conduct this study because of their unique properties that attracted much attention of researchers in the last decade [35, 36]. Indeed, CDs are easy to synthetize from small molecules and to functionalize. They are of very small size (a few nm) and fully water-soluble. In addition, they exhibit intrinsic fluorescence, that allows to study their cellular fate without prior derivatization with a fluorescent dye [37, 38]. Thus, CDs are currently developed for optical imaging applications, small drug or nucleic acid delivery, or theranostic applications [39–42]. In the present study, CDs were prepared as previously described [33, 43]. They were characterized in terms of size, hydrodynamic diameter, surface charge, but also surface charge density. Their uptake by human macrophages and airway epithelial cells was studied using confocal laser scanning microscopy (CLSM) and fluorescence activated cell sorting (FACS), thanks to their intrinsic fluorescence properties. Their cell toxicity was assessed by measuring viability loss, oxidative stress, inflammation, mitochondrial perturbation and lysosome integrity. We also investigated airway inflammation induced by the NPs in healthy mice, and their effects in a mouse model of asthma. Our data revealed that the surface charge density of a cationic carbon NP rather than the absolute value of its ζ-potential may be a relevant descriptor for predicting its toxicity.

## Results

### Physicochemical characterization of NPs

The elemental composition, ζ-potential, size, surface charge density, and optical properties of the 6 NPs were determined as described in Methods section. The nitrogen, carbon and hydrogen contents in the various CDs were in between 2.4 and 14.6%, 26.6 and 49.0%, and 6.1 and 8.5%, respectively (Table 1). These data however are difficult to interpret, as these particles were all titratable and contained sodium (NP1) or chloride (NP2 to NP6) counter ions in various amounts at pH 7.4. The five cationic carbon NPs (NP2 to NP6) exhibited similar ζ-potential in between + 20.6 and + 26.9 mV, whereas the negative NP (NP1) had a ζ-potential of − 38.5 mV (Table 1). The rather high ζ-potential, in absolute values, of the 6 NPs did translate into high colloidal stability in water as was observed for all the samples. The NP size (TEM) and hydrodynamic diameter (DLS) were measured in 1.5 mM NaCl pH 7.4, and ranged from 12.6 to 39.5 nm and from 7.2 to 43.9 nm, respectively (Table 1). TEM and DLS provided consistent results, in some cases (NP1, NP2, and NP4). In other cases, however, significant differences between size and hydrodynamic diameter were observed (NP3, NP5 and NP6). This might result either from the overestimation of populations of aggregates in DLS (according to Rayleigh’s approximation, intensity of scattering depends on the particle diameter raised to the sixth power), or arbitrary removal of NP aggregates in TEM micrographs before image processing (most of aggregates were assumed to form under capillary forces upon deposition and drying of the samples on the TEM grids). The surface charge density as determined by polyelectrolyte titration increased from 0.23 to 4.39 µmol/mg for NP2 to NP5 (Table 1), which did reflect the nature of the passivation reagent used in the preparation of the NPs. Indeed, the higher the nitrogen content of the passivation reagent, the higher the surface charge density of the resulting NPs. One exception was observed with bPEI25k-based NP6 which surface charge density was lower than that of bPEI600-based NP5 (Table 1). This is explained by the fact that NP6 was obtained by pyrolysis of a mixture of two parts of citric acid and one part of bPEI25k (by weight), whereas NP5 resulted from the pyrolysis of one part of citric acid with four parts of bPEI600. Thus, it could be expected that NP5 would display a higher density of charge than NP6, although the polyamine chains in bPEI25k are longer than those in bPEI600. Besides, when considering the DMEDA-based NPs, it appeared that PEGylation in NP2 resulted in a decrease in the density of charge by a three-fold factor when compared to NP3 (Table 1). Due to its hydrophilic nature, PEG chains grafted on the NPs generate a hydrated cloud with a large exclusion volume that sterically precludes the NP cationic charges from interacting with the negative charges of the titrant PAA. This is fully consistent with the picture of a hydrated steric barrier on the particle surface that has been introduced in the 1970s [44].

**Table 1:** Physicochemical characteristics of the citric acid-based NPs investigated herein

NP	Passivation reagent	ζ-potential (mV)	Size (nm)	Hydrodynamic diameter in saline (nm)	Aggregation in culture medium	Elemental composition			Surface charge density (Q_ek_)
						N (%)	C (%)	H (%)	(µmol/mg)
NP1	–	− 38.5 ± 1.9	28.5	24.7 ± 1.7	None	9.51	39.60	6.06	nd
NP2	DMEDA/PEG	+ 26.9 ± 1.6	16.3	17.7 ± 0.9	+	2.42	48.96	8.48	0.23
NP3	DMEDA	+ 21.0 ± 1.5	12.6	43.9 ± 2.4	++	12.77	35.64	6.99	0.78
NP4	PEHA	+ 20.6 ± 1.2	17.9	19.1 ± 2.0	None	12.32	26.89	7.35	1.35
NP5	bPEI600	+ 23.9 ± 2.0	39.5	10.1 ± 0.4	++	14.58	28.97	7.37	4.39
NP6	bPEI25k	+ 22.7 ± 0.1	19.1	7.2 ± 0.1	++	11.80	26.64	6.95	2.95

None: hydrodynamic diameter remaining less than 100 nm; +, partially aggregated (hydrodynamic diameter ranging between 100 and 500 nm); ++, highly aggregated (hydrodynamic diameter greater than 500 nm); –, not applicable; nd, not determined

In biological fluids, NPs tend to aggregate or/and agglomerate due to ionic strength, pH or protein adsorption [45]. To assess the behavior of the title NPs during in vitro experiments, DLS measurements were performed on NP dispersions prepared in complete RPMI-1640 or DMEM-F12 culture medium. The results were expressed as a score, as measured size was out of the apparatus measurement range for three NPs. NPs with size remaining less than 100 nm were considered as dispersed (score none). NPs with a diameter ranging between 100 and 500 nm were considered as partially aggregated (score +), and NPs with a diameter greater than 500 nm were designated as highly aggregated (score ++). NP1 and NP4 remained dispersed, whereas NP2, NP3, NP5 and NP6 tended to aggregate in both culture media. The aggregation of NP2, NP3, NP5 and NP6 was more or less pronounced. Due to PEGylation, NP2 was only partially aggregated, whereas NP3, NP5 and NP6 were highly aggregated (Table 1). Neither ζ-potential nor surface charge density data would have enabled to anticipate the aggregation behavior of the NPs in culture media.

Regarding optical properties, all the NPs displayed a UV–visible absorption spectrum with an absorption peak centered at 336–361 nm and extending to 600 nm, without noticeable structure (Table 2). A mass attenuation coefficient, *ε*_m_, was calculated at the maximum absorption (around 360 nm). While the *ε*_m_ value was close to ca. 2 L g^− 1^ cm^− 1^ for most NPs, NP3 and NP6 were exceptions. Indeed, NP3 displayed an especially high *ε*_m_ value of 8.80 L g^− 1^ cm^− 1^, as was attested by the deep brown color of the sample. At the opposite, NP6 sample was only faint colored, which translated into the low *ε*_m_ value. The NPs displayed various photoluminescence characteristics, as could be expected due to the various protocols carried out for their preparation, particularly with regard to the reaction temperature [43]. Thus, the excitation wavelength ranged from 351 to 522 nm, while the fluorescence emission wavelength was maximum at 437–512 nm, which is easy to use for in vitro imaging (*vide infra*). Also, it is worth to note that NP3 demonstrated up-conversion photoluminescence emission but that was not investigated in further detail.

**Table 2:** Photoluminescence properties of the NPs

	λ_max_ (nm)	*ε*_m_ (L g^− 1^ cm^− 1^)	λ_ex_ (nm)	λ_em_ (nm)
NP1	336	1.31	351	437
NP2	350	1.89	421	501
NP3	350	8.80	522	512
NP4	354	2.54	425	460
NP5	354	2.32	368	462
NP6	361	0.14	425	444

### Cytotoxicity of NPs towards macrophages and airway epithelial cells

**Fig. 1:**
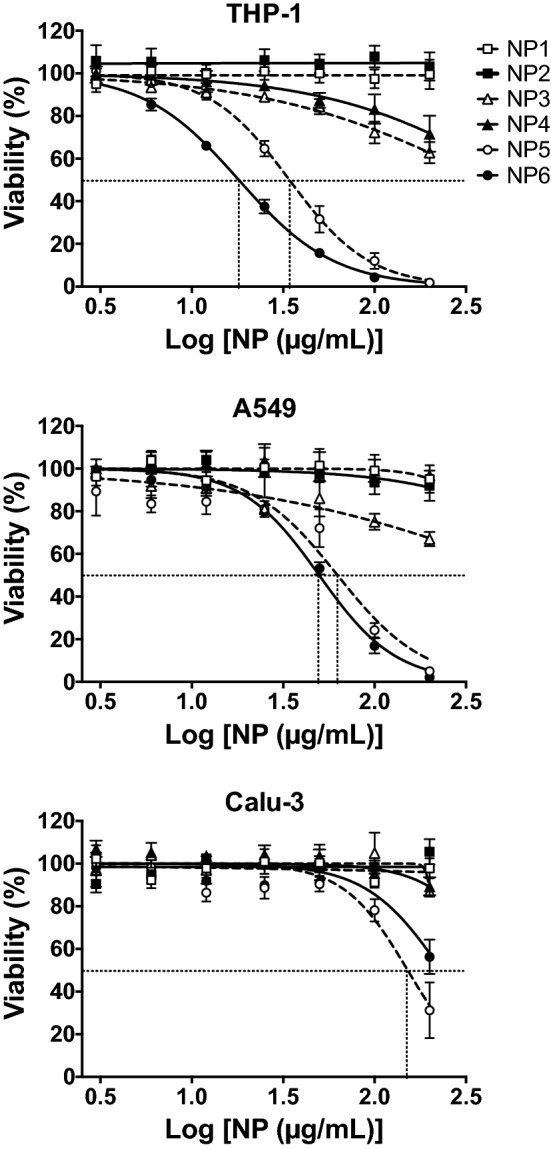
Cytotoxicity of the NPs in THP-1, A549 and Calu-3 cells. Cells were incubated or not with increasing concentrations (3–200 µg/mL) of the NPs for 24 h and their viability was assessed with the MTT assay. Results are expressed as percentage of viability when compared to control (unexposed cells). They are means ± SEM of n = 3–6 experiments. Concentration-response curves were obtained after logarithmic transformation of the data and fit with the Hill equation. Whenever possible, IC50 values were graphically determined (dashed lines)

**Fig. 2:**
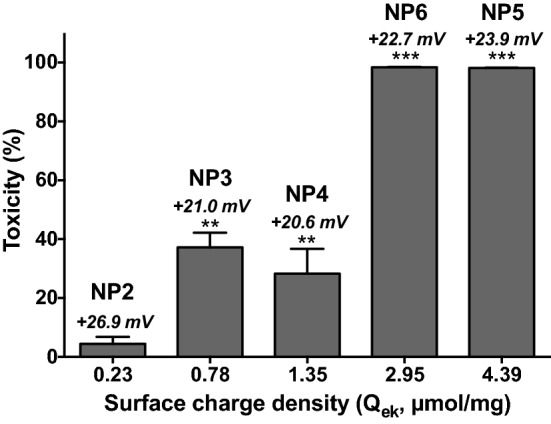
Cytotoxicity of NP1 to NP6 as a function of their surface charge density in THP1 cells. Cells were incubated with 200 µg/mL of the NPs for 24 h and their viability was assessed with the MTT assay. Results are expressed as percentage of viability loss when compared to control (unexposed cells). They are means ± SEM of n = 3–6 experiments. Statistical differences when compared to controls (CTL) were determined by one-way ANOVA followed by the Dunnett’s test. **p < 0.01 ; ***p < 0.001. ζ-potential of NPs are indicated above the bars

To assess NP cytotoxity, phorbol 12-myristate 13-acetate (PMA)-activated THP-1, A549 and Calu-3 cells were exposed to increasing concentrations of NP1 to NP6 (3 to 200 µg/mL) for 24 h, and their viability was determined by assessing cell mitochondrial activity using the MTT assay. The 6 NPs exhibited various toxicity grades in the different cell types tested. In THP-1 cells (Fig. 1), the high-(bPEI25k) and low-(bPEI600) molecular weight bPEI-passivated NPs, NP5 and NP6, with a ζ-potential of + 23.9 ± 2.0 and + 22.7 ± 0.1 mV, respectively, induced a significant and dose-dependent loss in cell viability that reached nearly 100% at the concentration of 200 µg/mL. NP3 and NP4 that were passivated with short oligoamines (DMEDA and PEHA, respectively) and exhibited a ζ-potential of + 21.0 ± 1.5 and + 20.6 ± 1.2 mV, respectively, triggered some toxicity at 100 and 200 µg/mL NPs, but viability loss did not exceed 37.2%. At last, the cationic CD NP2 that was passivated with DMEDA + mPEG550 (ζ-potential of + 26.9 ± 1.6 mV) and the negatively charged CD NP1 (ζ-potential of − 38.5 ± 1.9 mV) did not affect THP-1 cell viability. In A549 and Calu-3 cells, a similar trend was observed for the 6 NPs, but with less viability loss than in THP-1 cells for NPs exhibiting toxicity (Fig. 1). Indeed, in A549 cells, NP6 and NP5 induced a significant and dose-dependent loss in cell viability that reached nearly 100% at the concentration of 200 µg/mL, but with a higher IC_50_ value than in THP-1 cells: 62.7 (confidence interval (CI) 95% 49.4–79.4) and 49.7 (CI 95% 45.8–54.0) µg/mL in A549 cells vs. 34.2 (CI 95% 31.2–37.4) and 18.1 (CI  95% 16.9–19.2) µg/mL in THP-1 cells, for NP6 and NP5, respectively. In A549 cells as well, NP4 induced no significant viability loss in contrast to THP-1 cells. In Calu-3 cells, only NP6 and NP5 evoked some toxicity, but this toxicity did not exceed 43.6 and 68.7%, respectively at 200 µg/mL, when compared to nearly 100% in THP-1 cells for the two NPs. Thus, the present data confirmed our previous observation made on THP-1 cells [33] which was that the toxicity of cationic carbon NPs is not linked to the absolute value of their ζ-potential, since although exhibiting similar high ζ-potential, not all cationic NPs investigated herein triggered viability loss, whatever the cell model. Toxicity of the NPs appeared rather linked to their density of surface charge (Q_ek_). In the case of CDs, it is governed by the structure of the passivation reagent, the reagent stoichiometry, and the pyrolysis conditions. Thus, NP toxicity decreased with Q_ek_ as follow: NP6 (Q_ek_ = 2.95 µmol/mg) ≈ NP5 (Q_ek_ = 4.39 µmol/mg) > NP4 (Q_ek_ = 1.35 µmol/mg) ≈ NP3 (Q_ek_ = 0.78 µmol/mg) as depicted on Fig. 2 for THP-1 cells. Furthermore, the PEG decoration introduced at the surface of the NPs (compare NP2 to NP3) significantly decreased the density of the cationic charges (Q_ek =_ 0.78 µmol/mg for NP3 vs. 0.23 µmol/mg for PEGylated NP2) interacting with external components, which translated into lower toxicity (Fig. 2).

### NP cell uptake

**Fig. 3:**
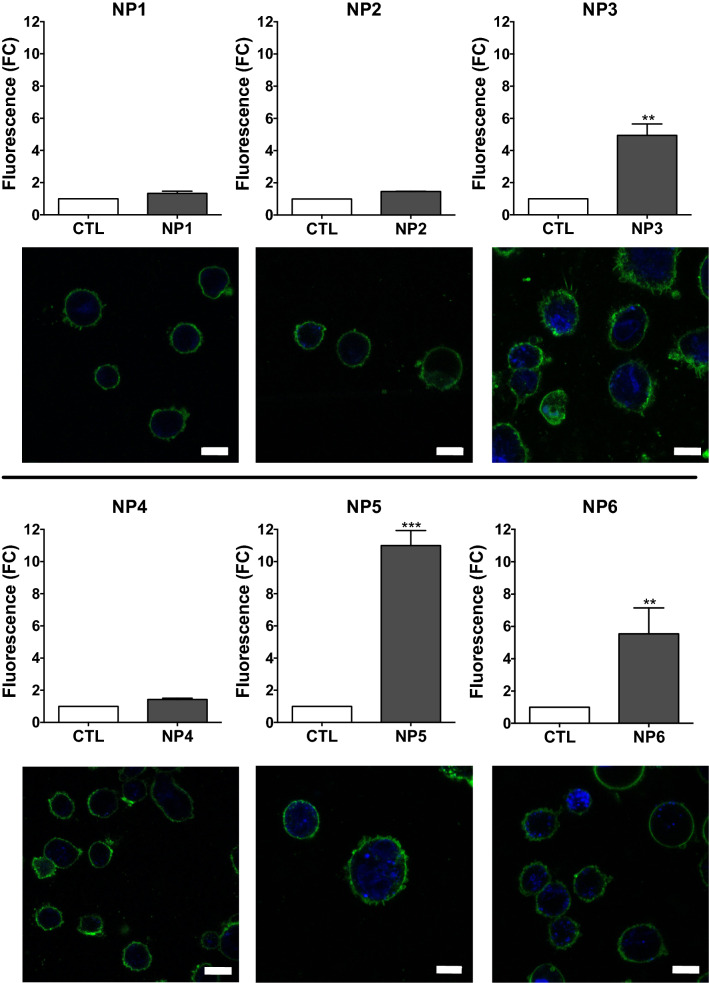
Uptake of NPs by THP-1 cells. **Graphs** quantification of NP internalization by FACS. Results are expressed as fold change in fluorescence intensity when compared to control cells (CTL). They are means ± SEM of n = 3 experiments. Statistical differences when compared to control were determined by one-way ANOVA followed by the Dunnett’s test. **p < 0.01; ***p < 0.001. **Pictures** CLSM of cells exposed to NPs. The cell membrane is colored in green thanks to the fluorescent probe DSQ12S and NPs appear in blue. All scale bars: 10 µm

NP surface charge is considered to impact NP toxicity by influencing NP cell uptake [24]. We thus assessed NP cell uptake by THP-1 and A549 cells using FACS and CLSM, thanks to the intrinsic fluorescence properties of CDs. Cells were exposed to 25 µg/mL NPs for 4 h before internalization measurements. FACS analysis showed a significant increase in fluorescence intensity in THP-1 cells treated with NP3 (4.9-fold, *p* < 0.01), NP5 (10.9-fold, *p* < 0.001) and NP6 (5.5-fold, *p* < 0.01) when compared to the control cells, suggesting internalization of these NPs. In contrast, no fluorescence increase was observed in cells exposed to NP4, NP2 and NP1 (Fig. 3). CLSM observations carried out after cell staining with the membrane probe DSQ12S (green fluorescence) confirmed the internalization of NP3, NP5 and NP6 measured by FACS (Fig. 3). Indeed, a blue labeling due to CD fluorescence was observed in THP-1 cells treated with NP3, NP5 or NP6, whereas no blue fluorescence was detected in cells exposed to NP4, NP2, and NP1. Similar data were obtained in A549 cells (Additional file 1: Fig. S1), with significant cell uptake being evidenced for NP3, NP5 and NP6 by both CLSM and FACS, although internalization appeared as less pronounced from FACS results except for NP3 (increases in fluorescence by 5.5- (*p* < 0.001), 3.1- (*p* < 0.001) and 2.4-fold (*p* < 0.01) for NP3, NP5 and NP6, respectively). Thus, internalization of the 6 NPs was overall consistent with their toxicity in both THP-1 and A549 cells, and was thus reflected by the surface charge density of the NPs rather than the value of their ζ-potential.

### Cellular responses evoked by the NPs

**Fig. 4:**
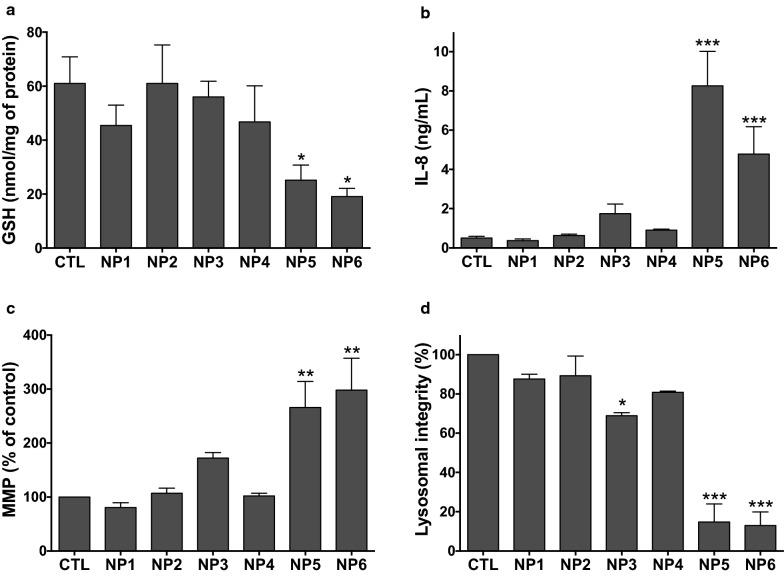
Cellular responses evoked by the NPs in THP-1 cells. Cells were exposed or not (CTL) to the NPs (100 µg/mL) for 4 h (**a** and **b**) or 24 h (**b** and **d**) and oxidative stress (**a**, reduced glutathione), inflammation (**b**, IL-8 secretion), mitochondrial perturbation (**c**, mitochondrial membrane perturbation–MMP) and lysosomal integrity (**d**, neutral red assay) were measured as described in Methods section. Data are means ± SEM of n = 3–6 experiments. They are expressed as percentage of controls (**c** and **d**) or absolute values (**a** and **b**). Statistical differences when compared to controls (CTL) were determined by one-way ANOVA followed by the Dunnett’s test. *p < 0.05; **p < 0.01 ; ***p < 0.001

Oxidative stress is a central mechanism of NP toxicity, including in the lung [46, 47]. Indeed, direct and/or indirect NP interaction and/or damage to cellular organelles such as lysosomes or mitochondria can lead to oxidative stress, which in turn evokes toxicological responses such as inflammation and viability loss. In a previous work, we found that cationic CDs passivated with high molecular weight bPEI evoked a dose–dependent viability loss that was associated with oxidative stress, IL-8 release, mitochondrial perturbation and loss in lysosome integrity in THP-1 cells [48]. Thus, to get further insight into the link between surface charge/charge density and cytotoxicity of cationic NPs, we investigated these cellular responses in THP-1 and A549 cells exposed to NP1 to NP6, as described in Methods section. NP5 and NP6 induced significant oxidative stress (*p* < 0.05 for the two NPs), IL-8 release (*p* < 0.001 for the two NPs), mitochondrial perturbation (*p* < 0.01 for the two NPs) and loss in lysosome integrity (*p* < 0.001 for the two NPs) in THP-1 cells (Fig. 4). Some increases in IL-8 secretion, mitochondrial perturbation and loss in lysosome integrity were also observed in response to NP3, but theses changes were significant for lysosome integrity only (*p* < 0.05). By contrast, NP2, NP4 and NP1 evoked no effect. In A549 cells, NP5 and NP6 induced some cellular responses in contrast to NP1 to NP4 (Additional file 1: Fig. S2). These effects related to oxidative stress and IL-8 secretion but not mitochondrial perturbation. Furthermore, they were of lower magnitude than in THP-1 cells, which is consistent with lower viability losses evoked by the NPs in A549 cells (Fig. 1).

As inflammation is an important in vitro and in vivo response to nanomaterials [5–12], an inflammatory cytokine multi-analyte ELISArray allowing the simultaneous assessment of 12 cytokines/chemokines was used to further characterize the inflammatory response evoked by the NPs (200 µg/mL) in THP-1 cells (Additional file 1: Table S1). Several cytokines (TNF-α, IL-6, IL-12, IL-17 and eotaxin) remained non detectable or in the background signal (i.e., similar to that recorded with non-exposed cells) after cell treatment with the various NPs. The release of MDC remained unchanged whatever the NPs the cells were exposed to. The two most toxic NPs, i.e. NP5 and NP6, increased by a 5- to 10-fold factor the release of IL-1β, MIP-1α, MIP-1β, and MCP-1. Although less pronounced (1.5- to 5-fold), some increase in MIP-1α and/or MIP-1β production was observed in cells exposed to NP3 and/or NP2. Besides, NP3 increased by more than 10-fold MCP-1 release. By contrast, no increase in the release of detectable cytokines was observed in response to NP1. Thus, IL-8 was not the only pro-inflammatory cytokine induced by the NPs that evoked cell toxicity.

All together, the cellular responses induced by the NPs in both THP-1 and A549 cells were overall consistent with their cell internalization (Fig. 3 and Additional file 1: Fig. S1) and the cell viability loss they triggered (Fig. 1), and better correlated with the surface charge density of the NPs, Q_ek_, than with their ζ-potential.

### Airway inflammation induced by the NPs in healthy mice

**Fig. 5:**
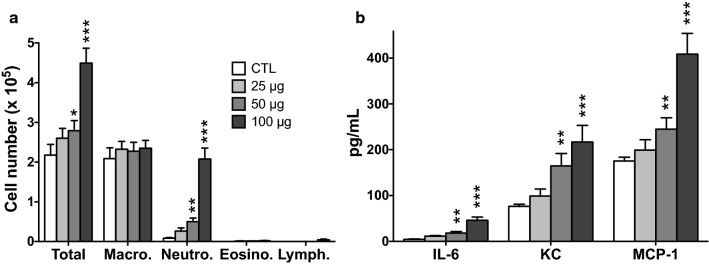
Dose-dependent airway inflammation induced by NP5 in mice. Mice received a single lung administration of 25, 50 or 100 µg of NP5 or saline (CTL), and airway inflammation was assessed 24 h later by counting cells (**a**) and measuring cytokines (**b**) in bronchoalveolar lavage fluids. Data are means ± SEM of n = 6 mice. Statistical differences when compared to controls (CTL) were determined by one-way ANOVA followed by the Dunnett’s test. *p < 0.05; **p < 0.01; ***p < 0.001

In toxicology, in vitro models may have limited reliability, mainly due to the fact that these models do not fully reflect the complexity of an organ or the interplay between different cell types or organs in the body [49]. To strengthen our in vitro results, we thus assessed inflammation induced by NP1 to NP6 in the lung of healthy mice. Animals received a single intrapulmonary administration of the NPs at fixed or increasing dose, and total and differential cells and inflammatory cytokines were measured in animal bronchoalveolar lavage fluids (BALFs) at 24 h. In a first experiment, we conducted a dose-response study on the two most toxic NPs in vitro, NP5 and NP6 (i.e., the bPEI-based NPs). NP5 induced a dose-dependent inflammation in the lung of mice. This inflammation was characterized by an influx of neutrophils and an increase in IL-6, KC and MCP-1 in BALFs (Fig. 5). It was non significant at the dose of 25 µg for all parameters, and maximal at the dose of 100 µg (*p* < 0.001 for all parameters). A similar dose-dependent inflammation was observed with NP6 (Additional file 1: Fig. S3).

**Fig. 6:**
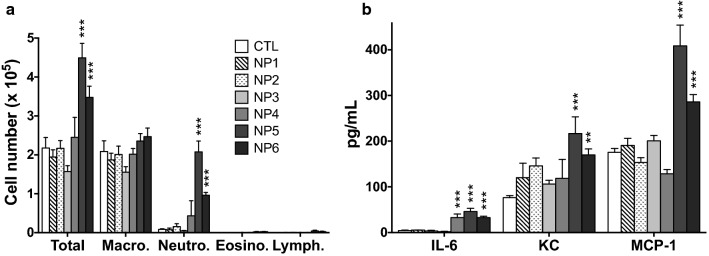
Airway inflammation induced by the 6 NPs in mice. Mice received a single lung administration of 100 µg of NPs or saline (CTL), and airway inflammation was assessed 24 h later by counting cells (**a**) and measuring cytokines (**b**) in bronchoalveolar lavage fluids. Data are means ± SEM of n = 6 mice. Statistical differences when compared to controls (CTL) were determined by one-way ANOVA followed by the Dunnett’s test. **p < 0.01; ***p < 0.001

In a second experiment, we compared the toxicity on the 6 NPs using the effective dose of 100 µg. NP5 and NP6 exhibited a significant inflammatory activity, as expected (Fig. 6). NP4 triggered a non-significant neutrophil influx and an increase in IL-6 (*p* < 0.01), whereas cell counts and inflammatory cytokine levels remained unchanged for NP1, NP2 and NP3. Thus, like in vitro data, in vivo results on lung toxicity support the hypothesis that toxicity of cationic carbon NPs is not linked to the absolute value of their ζ-potential. Lung toxicity of NPs rather decreased with Q_ek_, supporting the idea that surface charge density of a cationic carbon NP is a more relevant descriptor for predicting its in vitro and/or in vivo toxicity.

### Effects of the NPs in a mouse model of asthma

**Fig. 7:**
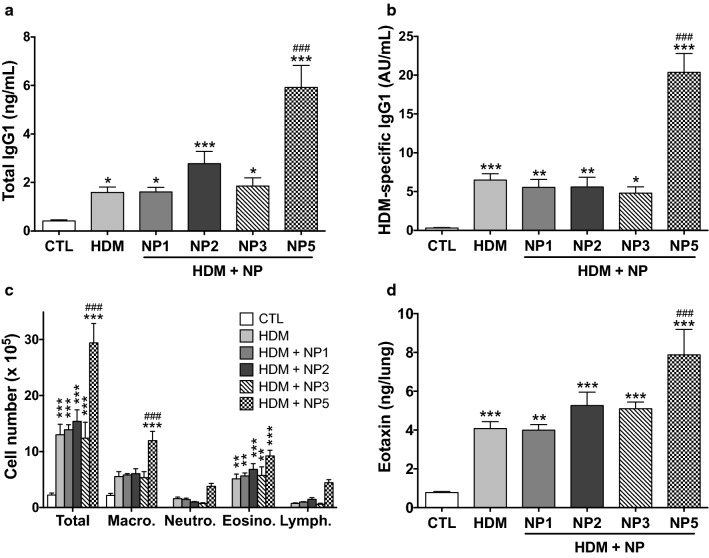
Effects of the NPs in a HDM-induced mouse model of asthma. Total (**a**) and allergen (HDM)-specific (**b**) IgG1 levels in serum of control mice (CTL) and mice exposed to allergen alone (HDM) or allergen and NP (HDM + NP). **c**: Total and differential cell counts in bronchoalveolar lavage fluids of mice. **d**: Eotaxin levels in lung homogenates of mice. Data are means ± SEM of n = 6 mice. Statistical differences were determined by one-way ANOVA followed by the Dunnett’s test. *p < 0.05; **p < 0.01; ***p < 0.001, when compared to control group. ^###^*p* < 0.001, when compared to HDM group

**Fig. 8:**
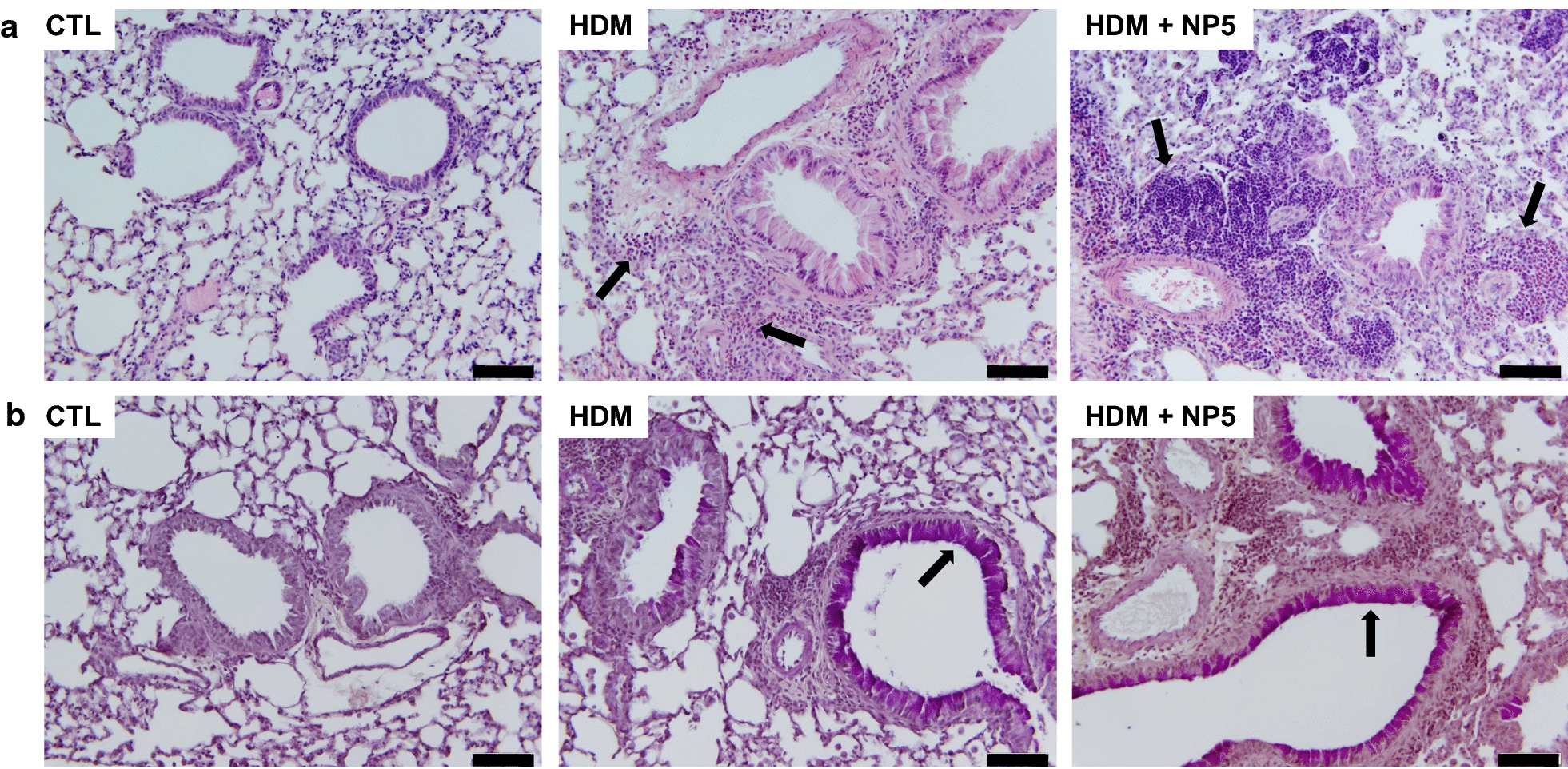
Effects of NP5 on allergen–induced tissular inflammation (**a**) and mucus production (**b**) in mice. Lung sections from control mice (CTL) and mice exposed to allergen alone (HDM) or allergen and NP5 (HDM + NP5) were stained with hematoxylin and eosin (**a**) or periodic acid-Schiff with counterstaining with HE (**b**). Arrows indicate cell infiltrate (**a**) or mucus staining (**b**). Scale bar = 50 µm

NP inhalation has been shown to promote allergic asthma, as evidenced by increased allergen sensitization and worsening of allergen-induced airway inflammation and remodeling in animal models of the disease [12–18]. So, in a last experiment, we compared the effect of NP1, NP2, NP3 and NP5 in a mouse model of allergic asthma induced by HDM, one of the main causes of the disease in humans [19]. HDM extract or vehicle was administered in the lung of mice on days 0, 7, 14 and 21 of the protocol and NPs (50 µg) were given every other day from day 0 to day 16. On day 23, total and HDM-specific IgG1 levels, total and differential cell counts and levels of the eosinophil chemoattractant eotaxin were measured in mouse serum, BALFs or lung homogenates. HDM alone significantly increased levels of total (*p* < 0.05) and allergen (HDM)-specific (*p* < 0.001) IgG1 in serum (Fig. 7a, b), total cell (*p* < 0.001) and eosinophil (*p* < 0.01) number in BALFs (Fig. 7c) and production of the eosinophil chemoattractant eotaxin (*p* < 0.001) in lung homogenates (Fig. 7d). NP5 aggravated all allergen-induced responses, with significant effects (*p* < 0.001) on total and allergen-specific IgG1 in serum, total cell and macrophage number in BALFs, and production of the eosinophil chemoattractant eotaxin in lung homogenate when compared to the allergen (HDM) group. On the other hand, NP1, NP2 and NP3 did not exacerbate any allergen response. To extend the results obtained on NP5, histological analysis was conducted on mouse lung sections to evidence changes in tissular inflammation and mucus production. Perivascular and peribronchial inflammatory cell infiltrate (Fig. 8a) and increased mucus production (Fig. 8b) were observed on lung sections of allergen-exposed mice (HDM) when compared to control mice (CTL), and these changes (Fig. 8a, b) were more pronounced on sections from allergen-treated mice exposed to the NP (HDM + NP5).

All together, like in healthy mice, toxicity of cationic carbon NPs in allergen-sensitized animals appeared dependent on their surface charge density, i.e. the nature of their surface passivation, rather than on the absolute value of their ζ-potential.

## Discussion

To investigate the role of the surface charge in the toxicity of cationic NPs, we synthesized and assessed the in vitro and in vivo lung toxicity of 5 cationic carbon NPs displaying various amounts of amino groups at their periphery. A non-passivated and non-toxic anionic NP was added to this NP library as control. Our data demonstrate that the surface charge density of a cationic carbon NP rather than the absolute value of its ζ-potential is a relevant descriptor for predicting its toxicity.

The NPs that were prepared in the present study are spherical carbon nanomaterials called CDs that possess intrinsic fluorescence among other interesting properties. A large number of methods have been described for the synthesis of these NPs and have been reviewed in the recent literature [50–55]. Especially, bottom-up methods producing CDs by thermal treatment of organic precursors, generally a carbon source and a passivation reagent, became very popular as they use low cost starting materials and do not require any sophisticated equipment. Though it is widely acknowledged that intrinsic properties of CDs depend on the experimental conditions carried out for their preparation, the latter are extremely difficult to anticipate, and their fine tuning still relies on protocols to implement step by step, through a trial and error approach. Herein, several independent protocols have been selected in order to produce cationic CDs with various charge distributions at their periphery. We thus varied the carbon source (citric acid and ammonium citrate), passivation reagent (DMEDA, PEHA, bPEI600, bPEI25k, and mPEG550), and activation mode (microwave irradiation, thermal decomposition, and solvothermal treatment). The obtained cationic NPs exhibited very similar ζ-potential (in between + 21.0 and + 26.9 mV), but different surface charge density (from 0.23 to 4.39 µmol/mg), which allowed us to investigate the hypothesis that the amount of positive charges on NPs rather than the ζ-potential value is predictive of NP toxicity.

In the first part of our study, human macrophages (PMA–activated THP-1 cells) and airway epithelial cells (A549 and Calu-3 cells) were used to assess the *in vitro* toxicity of our NP library. These two cell types were selected as they are the two main potential targets of NPs in the lung [3, 12]. PMA–activated THP-1 cells are widely used as a model of human macrophages in nanotoxicology [56]. We and others used this model to screen a large library of carbon or metal NPs with different physicochemical characteristics [5, 33, 57, 58]. Besides, A549 and Calu-3 cells are epithelial cells representative of the two main regions of NP deposition in the airways, namely the alveoli and the bronchi, respectively. They are also among the most commonly used lung epithelial cell lines in nanotoxicology studies [49]. Our data show that although exhibiting similar ζ-potential, not all cationic NPs of our library were internalized and triggered viability loss and/or NP-associated cell responses including oxidative stress, cytokine secretion, mitochondrial perturbation and lysosome integrity changes. Consistent data among the different toxicity endpoints we measured were obtained for all NPs. Thus, we confirmed our previous observation made in macrophages that cytotoxicity of cationic carbon NPs is not necessarily linked to the absolute value of their ζ-potential [33] and extended this observation to airway epithelial cells. The two tested epithelial cell lines were however less sensitive to the toxicity of the cationic NPs than macrophages. This is in agreement with previous reports in the literature on metallic or PLGA NPs [5, 59]. The difference in sensitivity observed between macrophages and epithelial cells could be explained by the phagocytosis activity of macrophages and their major role in particle clearance in contrast to alveolar epithelial cells. Besides, among the two epithelial cell lines investigated herein, Calu3 cells appeared as less sensitive than A549 cells. This could be explained by the capacity of Calu-3 cells to secrete mucus that acts as an important protective barrier at epithelial interfaces and may thus reduce NP cell uptake [59]. In agreement with this hypothesis, uptake of polystyrene particles by Calu-3 cells was reported to be reduced when compared to A549 cells [60, 61].

In nanotoxicology, the relevance of in vitro studies to predict in vivo nanomaterial toxicity has been questioned because these models do not fully reflect the complexity of an organ or the interplay between different cell types within an organ, but also for more specific reasons [49]. Indeed, some in vitro assays widely used for toxicity assessment of chemicals have been reported to generate false results with some NPs because these nanomaterials interfered with chemical or fluorescent probes [6, 62]. As well, in biological fluids, NPs may adsorb biomolecules such as proteins and/or lipids due to their large specific surface area. These biomolecules, which alter the surface chemistry of the NPs and therefore their interactions with cells and their toxicity, may be different in in vitro and in vivo models, due to difference in microenvironment composition [23]. However, in this study, in vivo data reinforce the observation made in vitro that toxicity of cationic carbon NPs is not a reflection of the absolute value of their ζ-potential, since only cationic NPs that exhibited significant in vitro toxicity, namely NP5 and NP6, triggered airway inflammation in healthy mice and/or exacerbated systemic immune response, and airway inflammation and mucus production in mice exposed to an allergen. Thus the in vitro models we selected were rather predictive of the in vivo lung toxicity of our NPs, especially THP-1 and A549 cells in which NPs exhibiting potent in vivo toxicity triggered significant changes in several toxicity endpoints, particularly oxidative stress which plays a central mechanistic role in NP-induced toxicity, including in the lung [19, 46, 47].

ζ-potential is widely used to characterize the charge of NPs and to predict NP toxicity, cationic NPs being generally more toxic than anionic ones, due in part to their greater cell uptake and/or their damaging effect on cell and lysosomal membranes [25–27, 48, 63]. A correlation was thus found between ζ-potential and hemolytic activity and lung inflammogenicity of polymeric or polystyrene NPs [28, 29]. However, not all studies found a correlation between ζ-potential of NPs and cytotoxicity [64, 65]. By screening a library of 35 carbon-based NPs with various surface functionalization in human macrophages, we found that a cationic charge is not sufficient to confer toxicity to NPs [33]. The cationic NPs in this library were produced by pyrolysis of organic materials, in the presence of nitrogen-containing passivation reagents with an increasing number of amino groups, though this did not strictly translate into increasing ζ-potential values. ζ-potential is defined as the average electrostatic potential existing at the slipping plane (i.e., the boundary plane delimitating the NP and associated counter ions that move together when an electrical field is applied) [66]. Thus, whether the slipping plane is close to the particle surface or not, the surface charge density determined by the amount of charged functional groups (ammonium) tethered to the NP within the electrical double layer (delimited by the surface of the NP core and the slipping plane) may vary a lot when the ζ-potential value may be the same. Therefore, the amount of positive charges on NPs (surface charge density), rather than the ζ-potential value could be a better predictor of NP toxicity. In agreement with our hypothesis, in their review on how physicochemical characteristics of NPs cause their toxicity, Luyts et al. came to the conclusion that the charge at specific spots contributes significantly to the NP toxicity, making ζ-potential not a safe predictor of nanotoxicity [20]. However, NP charge density has not been investigated as a descriptor of NP safety in the literature. The determination of the surface charge density (Q_ek_) of cationic NPs is especially difficult and cannot be achieved by standard acid-base titration. Indeed, due to high local concentration of amino groups at the surface of cationic NPs, their apparent p*K*_a_ span over a wide pH range that can extend from 11 to 2 [67, 68]. In the present study, we thus used polyelectrolyte titration to determine the charge density of our cationic NPs [69, 70]. We found thereby that toxicity of the cationic carbon NPs decreased with Q_ek_ in both in vitro and in vivo lung models whatever the measured endpoints as summarized in Table 3. The investigated NPs appeared to rank in 3 groups according to their toxicity. The first group includes the non toxic PEGylated DMEDA-passivated CDs, NP2, that displays the lower Q_ek_ value in the series. In the second group, we find NPs exhibiting low in vitro toxicity and no *in vivo* toxicity, i.e. NP3 (DMEDA-passivated CDs) and NP4 (PEHA-passivated CDs). These NPs are characterized by low/medium Q_ek_. NP5 (bPEI600-passivated CDs) and NP6 (bPEI25k-passivated CDs) that triggered both significant in vitro and in vivo toxicity are arranged/classified in the third group, and display the higher Q_ek_ values in the series. This may suggest the existence of a threshold surface charge leading to harmful lung effects.

**Table 3:** Summary of the charge characteristics and in vitro and in vivo toxicity of the NPs investigated herein

	NP1	NP2	NP3	NP4	NP5	NP6
**Charge characteristics**						
ζ-potential (mV)	− 38.5 ± 1.9	+ 26.9 ± 1.6	+ 21.0 ± 1.5	+ 20.6 ± 1.2	+ 23.9 ± 2.0	+ 22.7 ± 0.1
Q_ek_ (µmol/mg)	nd	0.23	0.78	1.35	4.39	2.95
**In vitro toxicity in THP-1 cells**						
Viability loss	–	–	**	**	***	***
Oxidative stress	–	–	–	–	*	*
IL-8	–	–	–	–	***	***
Mitochondrial perturbation	–	–	–	–	**	**
Lysosome integrity	–	–	*	–	***	***
**In vivo toxicity in healthy mice**						
Total cells	–	–	–	–	***	***
Neutrophils	–	–	–	–	***	***
IL-6	–	–	–	***	***	***
KC	–	–	–	–	***	**
MCP-1	–	–	–	–	***	***
In vivo toxicity in allergic mice						
Total IgG1	–	–	–	nd	***	nd
HMD-specific IgG1	–	–	–	nd	***	nd
Total cells	–	–	–	nd	***	nd
Eosinophils	–	–	–	nd	***	nd
Eotaxin	–	–	–	nd	***	nd

nd: not determined

–: no significant effect

*: significance of the biggest effect evoked by the NPs compared to control (*p < 0.05; **p < 0.01; ***p < 0.001)

## Conclusions

All together, using both in vitro and in vivo models, this study clearly reveals that the surface charge density of a cationic carbon NP rather than the absolute value of its ζ-potential may be a relevant descriptor for predicting lung toxicity. In the case of CDs, surface charge density is governed by the nature of the passivation reagent, the reagent stoichiometry, and the pyrolysis conditions. Whether our observation may apply to other kinds of NPs and other toxic responses, and by the way, whether surface charge density can be an important feature to better predict cationic NP safety deserves deeper investigations.

## Methods

### Preparation of NPs

The NPs investigated herein were produced according to some previously reported protocols [33, 43]. They were purified by extensive dialysis and were obtained in 10–44% yield.

NP1. Triammonium citrate (5.00 g) and potassium phosphate monobasic (1.00 g) in pure water (10 mL) were mixed to homogeneity in an Erlenmeyer flask and heated in a domestic microwave oven for 120 s at 700 W (i.e., under normal pressure). The resulting glassy residue was resuspended in EtOH (50 mL) and refluxed under stirring for 4 h. The suspension was then cooled down and stored at 4 °C overnight for decantation. Filtration of the supernatant through a 0.22 µm polyethersulfone (PES) membrane and solvent removal under reduced pressure yielded NP1 (0.70 g).

NP2. Neat citric acid (0.50 g), mPEG550 (4.30 g), and DMEDA (1.00 g) were heated at 150 °C, under normal pressure, and volatile was integrally driven out of the reaction vessel. After 30 min, the temperature was raised to 230 °C and the reaction mixture was stirred at this temperature for 30 min. The resulting residue was cooled down to rt, dissolved in water and dialyzed (Spectra/Por 3, MWCO 1000 Da) for 24 h. The resulting brown solution was filtered through a 0.22 µm PES membrane and freeze-dried to yield NP2 as a hygroscopic powdered dark brown material (0.64 g).

NP3. These NPs (2.40 g) were obtained from citric acid (6.00 g) and DMEDA hydrochloride (11.31 g) using the same protocol as for NP2.

NP4. These NPs (5.63 g) were obtained from citric acid (6.00 g) and PEHA (21.70 g) using the same protocol as for NP2.

NP5. Citric acid (125 mg), bPEI600 (500 mg), and HCl 0.1 N (5 mL) were homogenized in an Erlenmeyer flask, then heated in a domestic microwave oven at 620 W for 170 s. The residue was dissolved in HCl 0.1 N, centrifuged (7,500 g, 5 min), and supernatant was loaded in a dialysis bag (MWCO 3,500 Da) for extensive dialysis against HCl 0.1 N (24 h) and ultra pure water (24 h). Freeze-drying of the dialysis bag content yielded NP5 (275 mg) as a brown hygroscopic powder.

NP6. Citric acid (5.00 g) and bPEI25k (2.50 g) in water (50 mL) were stirred under reflux for 24 h. The mixture was cooled to rt, centrifuged (7500*g*, 5 min), and supernatant was loaded in a dialysis bag (MWCO 3500 Da) for extensive dialysis against HCl 0.1 N (96 h) and ultra pure water (24 h). Freeze-drying of the dialysis bag content yielded NP6 (0.75 g) as a brown hygroscopic powder.

### Characterization of NPs

The elemental composition of the NPs was determined by analysis on a Vario EL III instrument (Elementar) and expressed as carbon (C), hydrogen (H), and nitrogen (N) mass content (%). The size of the NPs was determined by transmission electron microscopy (TEM). CD samples (0.5 µL, 1.0 mg/mL in 1.5 mM NaCl pH 7.4) were deposited on glow-discharged (90 V and 2 mA for 15 s) carbon-coated grids (Cu-300HD, Pacific Grid Tech). After at least 2-hour drying at rt, the grids were observed using a bench top LVEM5 microscope (Delong Instrument) operating at 5 kV. The average size of the NPs was determined by image analysis using the ImageJ software (v 1.50i, NIH), from a set of 300–1000 particles. The hydrodynamic diameter and ζ-potential value of NPs were measured by dynamic light scattering (DLS) on a Zetasizer NanoZS apparatus (Malvern Instruments). Measurements were performed in triplicate on fresh samples (1.0 mg/mL in 1.5 mM NaCl pH 7.4) at 25 °C. Data were analyzed using the multimodal number distribution software supplied with the instrument and expressed as mean (± SD). DLS was also used to assess the aggregation of NPs in culture medium. NP dispersions were prepared at 1.0 mg/mL in complete RPMI-1640 or DMEM-F12 containing 10% fetal serum, and measurements were performed extemporaneously without sonication of the dispersions. The surface charge density of NPs was determined by means of polyelectrolyte titration [69, 70], monitoring ζ-potential variation of CDs (1.0 mg/mL, NaCl 1.5 mM pH 7.4) along spiking with a solution of poly(acrylic acid) (PAA, MW ± 1,800 Da, NaCl 1.5 mM pH 7.4). The amount of PAA required to reverse the sign of ζ-potential was approximated by linear interpolation of the titration curve at the isoelectric point. The density of surface charge (electrokinetic charge, Q_ek_) of the NPs was then calculated from the amount of required PAA by use of equation:

$$Q_{{ek}} = V \cdot c/w$$

where V is the volume of titrant added (µL), c the concentration of the acrylic acid titrant (µmol_AA_/µL), and w the amount of titrated NPs (mg). The results were thus expressed in mmol/g. Approximating the density of CDs at 1, the amount of charge per particle (C/particle) and per surface unit (C/nm^2^) were also calculated. To characterize optical properties of the NPs, NP samples (0.10 mg/mL) were prepared in ultra-pure water and UV-visible and fluorescence measurements were done using a UviKon XL spectrometer (Bio-Tek Instruments) and a Fluoromax-4 spectrofluorometer (Horiba Scientific) respectively, in a 1-mL quartz cuvette.

### Cell culture

THP-1 (TIB-202™), A549 (CCL-185TM) and Calu-3 (HTB-55TM) cells were grown in culture flasks at 37 °C in a 5% CO_2_ humidified chamber. RPMI-1640 culture medium containing Lglutamine (2 mM), 2-mercaptoethanol (0.05 mM), penicillin (100 UI/mL), streptomycin (100 µg/mL), and heat inactivated fetal bovine serum (10%) was used to cultivate THP-1 cells. A549 and Calu-3 cells were grown in DMEM/F12 culture medium containing L-glutamine (2 mM), penicillin (100 IU/mL), streptomycin (100 µg/mL), Hepes (5 mM) and fetal bovine serum (10%). All cells and culture reagents were from ATCC and GIBCO, respectively. The day prior experiments, cells were transferred into culture plates or IbiTreat® µ-Slides (1.5 polymer coverslip, IBIDI), as described below.

### Assessment of changes in cell viability evoked by the NPs

Changes in cell viability evoked by the NPs were assessed in THP-1, A549 and Calu-3 cells using the MTT assay. Cells were seeded into 96-well culture plates at a density of 3.10^4^ (A549) or 10^5^ (THP-1 and Calu-3) cells/well. PMA (Sigma, 10 ng/mL) was added to culture medium of THP-1 cells to induce their differentiation into macrophages. The following day, all cells were incubated with increasing concentrations of NPs (3–200 µg/mL) for 24 h. Then, cells were carefully washed with phosphate buffered saline (PBS) before addition of MTT (100 µL, 1.0 mg/mL in complete culture medium, Sigma). After a 1-h incubation period, culture medium was removed and cells were lysed with DMSO. Absorbance of the resulting samples was read at 570 nm with a correction at 690 nm using a Multiskan FC reader (Thermo Scientific). Cell viability was expressed as the percentage of the absorbance of treated cells relative to the absorbance of the non-exposed control cells. Concentration-response curves were obtained after logarithmic transformation of the data and fit with the Hill equation. Then, the Hill equation was used to calculate the effective concentration triggering 50% (EC_50_), when possible.

### Assessment of NP cell uptake

NP cell uptake was assessed in THP-1 and A549 cells by CLSM and FACS, thanks to the intrinsic fluorescence properties of CDs. CLSM experiments were carried out as previously described [48]. Briefly, THP-1 and A549 cells were seeded into 8-well IbiTreat µ-Slides at a density of 10^5^ and 75.10^3^ cells/well, respectively. PMA (10 ng/mL) was added to culture medium of THP-1 cells to induce their differentiation into macrophages. The following day, the different cultures were incubated with 25 µg/mL NPs for 4 h. At the end of the incubation time, cells were carefully washed with culture medium to remove non-internalized NPs and the DSQ12S fluorescent probe (10 nM in PBS) was added to the samples for 5 min to label the cell membrane [71]. The intracellular distribution of NPs was then observed using a Leica SP2 microscope equipped with a 63× oil immersion objective (NA = 1.2). The NPs and the membrane probe were excited with 405 and 635 nm laser sources, respectively. The emission bands were detected with a photomultiplier. The position and the width of the detection channels were adjusted for each dye.

For FACS experiments, cells were seeded into 24-well plates at a density of 18.10^4^ (A549) or 5.10^5^ (THP-1) cells/well. PMA (10 ng/mL) was added to culture medium of THP-1 cells to induce their differentiation into macrophages. The following day, the different cultures were exposed to 25 µg/mL NPs for 4 h. At the end of the exposure period, the cells were washed with PBS, detached with trypsin, centrifuged and resuspended in 500 µL of culture medium without serum. Samples were then analyzed on a LSRFortessa X-20TM cytometer (BD Biosciences) after sample excitation at 405 nm and emission signal detection at 450 nm. Data were analysed with the FlowJo software. Very small and very granular events (cell debris) were excluded from the analysis. For A549 cells, cell doublets (approximately 20% of the events identified on FSC-H/SSC-W cytograms) were also excluded.

### Oxidative stress assessment

Oxidative stress was assessed by measuring changes in cellular reduced glutathione (GSH) induced by NPs in THP-1 and A549 cells using the naphthalene-2,3-dicarboxaldehyde (NDA) probe. Cells were seeded into 24-well culture plates at a density of 18.10^4^ (A549) or 5.10^5^ (THP-1) cells/well. PMA (10 ng/mL) was added to culture medium of THP-1 cells to induce their differentiation into macrophages. The following day, the cells were incubated with 100 µg/mL NPs for 4 h. At the end of the incubation period, cells were washed with a buffer containing 5 mM EDTA, 40 mM NaH_2_PO_4_, 110 mM Na_2_HPO_4_, pH 7,4 and lysed with 0.1% Triton X100®. Then, proteins were denatured and precipitated with 0.1M hydrochloric acid and 50% sulfosalicylic acid, before sample centrifugation (10,000*g*, 15 min, 4 °C). Cell lysates were then incubated with the NDA probe for 25 min at 4 °C, before fluorescence measurement (λ_ex_ = 485 nm; λ_em_ = 528 nm, Varioskan™ LUX reader, Thermo Scientific). A calibration curve was used to calculate the amount of reduced GSH in the samples. This amount was then expressed in nmol of GSH per mg of protein. To do so, protein concentration in cell lysates was determined using the bicinchoninic assay according to the manufacturer’s instructions. All reagents were from Sigma.

### Mitochondrial membrane potential assay

Mitochondrial membrane potential was assayed in THP-1 and A549 cells using the JC-10 fluorescent probe (Sigma). Cells were seeded into 96-well culture plates at a density of 3.10^4^ (A549) or 10^5^ (THP-1) cells/well. PMA (10 ng/mL) was added to culture medium of THP-1 cells to induce their differentiation into macrophages. The following day, all cells were incubated with 100 µg/mL NPs for 4 h. Then, the cell culture supernatant was removed and the JC-10 probe (100 µL) was added to the cells for 1 h. Fluorescence of the samples was then measured (functional mitochondria: λ_ex_ = 540 nm, λ_em_ = 590 nm; non functional mitochondria: λ_ex_ = 490 nm, λ_em_ = 525 nm), and the ratio of fluorescence intensity at 525 nm to fluorescence intensity at 590 nm was calculated for each sample. Then, data were expressed as the percentage of fluorescence of NP-exposed cells relative to the fluorescence ratio of non-exposed control cells.

### Neutral red assay

Neutral red (NR, Sigma) assay was used to assess lysosomal membrane integrity after exposure of THP-1 or A549 cells to NPs. Cells were seeded into 96-well culture plates at a density of 3.10^4^ (A549) or 10^5^ (THP-1) cells/well. PMA (10 ng/mL) was added to culture medium of THP-1 cells to induce their differentiation into macrophages. The following day, the cells were exposed to 100 µg/mL NPs for 24 h. At the end of the incubation period, culture medium was removed and cells were carefully washed with PBS. Complete culture medium containing NR (200 µL of a 100 µg/mL solution) was added to the cells for dye incorporation into intact lysosomes. After a 3-h incubation period, culture medium was removed and cells were lysed with 1% acetic acid solution containing 50% ethanol to release the incorporated dye. Absorbance of the resulting samples was read at 570 nm with a correction at 690 nm. Results were expressed as the percentage of the absorbance of treated cells relative to the absorbance of the non-exposed control cells.

### Animals

Nine-week-old male Balb/c mice were purchased from Charles River Laboratories. They were housed in polycarbonate exhaust ventilated cages with bedding made from spruce wood chips. The animal room was maintained under controlled environmental conditions (temperature of 20 ± 2 °C, relative humidity of 50 ± 10% and 12 h/12 h light/dark cycle). Food and tap water were available ad libitum. The animals were acclimated for 1 week before the initiation of the study. Animal experiments were conducted in compliance with the European legislation (Directive 2010/63/EU). Experimental protocols were approved by the local ethics committee (CREMEAS) under the agreement number #4674.

### Exposure of healthy mice to NPs

Healthy mice received one intrapulmonary administration of NPs at increasing (10, 50 and 100 µg) or fixed (100 µg) doses and were used 24 h later. NPs were administered by intranasal instillation of 25 µL of a NP solution prepared in saline. Instillations were carried out under anaesthesia (50 mg/kg ketamine (Imalgen®, Merial) and 3.33 mg/kg xylazine (Rompun®, Bayer) given *i.p.*). Control animals received instillations of the same volume of saline alone.

### Study of NPs in a mouse model of asthma

A house dust mite (HDM) model of asthma was used as previously described [19]. Mice were divided into six groups: a group that received the vehicle alone (control group), a group that received HDM alone (HDM group), and four groups that received HDM + NP1, NP2, NP3 or NP5 (HDM + NP groups). HDM extract (*Dermatophagoides pteronyssinus* extract, 2 µg Der p 1/administration, GREER® Laboratories Inc.) or vehicle was administered to mice on days 0, 7, 14 and 21 of the protocol. NPs (50 µg/administration) were administered every other day from day 0 to day 16. All animals were used on day 23. HDM extract, NPs or their vehicle were administered in the lung of mice by intranasal instillation of a saline solution (25 µL/administration) containing HDM alone, NPs alone or HDM + NP. Instillations were carried out as described above.

### Serum, bronchoalveolar lavage fluid and tissue collection and processing

The experiment was terminated by *i.p.* injection of a lethal dose of ketamine (150 mg/kg) and xylazine (10 mg/kg). Blood was drawn from mice by vena cava puncture, and collected serum was stored at − 80 °C until immunoglobulin measurements. After tracheotomy, the lungs were lavaged by 6 instillations of 0.5 mL ice-cold saline supplemented with 2.6 mM EDTA (saline-EDTA). Bronchoalveolar lavage fluids (BALF) recovered from the two first instillations were centrifuged (200*g* for 5 min at 4 °C) and the resulting supernatant was stored at − 20 °C until cytokine measurements. Cell pellets recovered from the 6 instillations were resuspended in saline-EDTA and used to determine total and differential cell numbers. After BALF collection, lungs were perfused *in situ* through the pulmonary artery with ice-cold PBS, collected and either frozen in liquid nitrogen and stored at − 80 °C until lung homogenate preparation and assays, or fixed in 4% paraformaldehyde for histology. Lung homogenates were prepared by homogenizing frozen tissue in 2 mL of PBS containing a protease inhibitor cocktail (Complete EDTA-free tablets, Roche) using an Ultra-Turrax® homogenizer (T25, Ika).

### Immunoglobulin assay in serum

Serum levels of total and HDM-specific IgG1 were determined by ELISA. Briefly, microtiter plates were coated with an anti-mouse IgG1 antibody (0.2 µg/well in PBS, pH 7.4, BD Biosciences) or HDM extract (0.25 µg Der p 1/well in 0.1 M bicarbonate buffer, pH 9.6) and blocked with PBS containing 1% bovine serum albumin (BSA). Serums diluted in PBS containing 1% BSA were then incubated overnight at 4 °C. Next, the plates were incubated with a biotinylated anti-mouse IgG1 antibody (BD Biosciences), an extravidin-horseradish peroxydase (Sigma-Aldrich) and the horseradish peroxydase substrate tetramethylbenzidine (TMB, BD Biosciences), successively. Sample absorbance was measured at 450 nm.

### Determination of total and differential cell counts in bronchoalveolar lavage fluids

BALFs were centrifuged (200*g* for 5 min at 4 °C) to pellet cells and erythrocytes were lysed by hypotonic shock. Cells were then resuspended in 500 µL ice-cold saline-EDTA and total cell counts were determined using a Neubauer’s chamber. Differential cell counts were assessed on cytologic preparations obtained by cytocentrifugation (Cytospin 4, Thermo Scientific) of 200 µL of diluted BALFs (250,000 cells/mL in ice-cold saline-EDTA). Slides were stained with Microscopy Hemacolor® (Merck) and at least 400 cells were counted for each preparation. Eosinophil, neutrophil, lymphocyte and macrophage numbers were then expressed as absolute numbers from total cell counts.

### Cytokine assays

Cytokines were quantified in culture supernatants of THP-1 and A549 cells by ELISA (IL-8) or a Multi-Analyte ELISArray (tumor necrosis factor-α (TNF-α), IL-1β, IL-6, IL-12, IL-17A, IL-8, monocyte chemoattractant protein 1 (MCP-1), regulated on activation normal T cell expressed and secreted (RANTES), macrophage inflammatory proteins MIP-1α and MIP-1β, macrophage-derived chemokine (MDC) and eotaxin), according to instructions of the manufacturer (R&D Systems for the ELISA and Qiagen for the ELISArray). Eotaxin was measured in lung homogenates of mice by ELISA, according to the manufacturer’s instructions (R&D Systems). In the ELISA assays, a calibration curve was used to calculate cytokine concentrations, expressed in pg/mL. In the ELISArray, absorbance values were expressed as fold change compared to the positive or negative control.

### Histology

Fixed lungs were rinsed in PBS, dehydrated and embedded in paraffin using standard procedures. Tissue sections (5 µm) were prepared and stained with hematoxylin and eosin (H&E) for morphologic assessment, and Periodic Acid-Schiff (PAS) for mucus visualization, respectively.

### Presentation and statistical analysis of the data

Data were plotted as concentration-response curves or bar charts and were analyzed with the GraphPad Prism 6.0 software. In the bar chart representations, statistical differences between groups were determined by one or two-way ANOVA followed by the Dunnett’s test. Data were considered as significantly different when *p* value was less than 0.05.

## Supplementary Information

**Additional file: Figure S1.** Cell uptake of NPs by A549 cells. **Table S1.** Cytokine production evoked by the NPs in THP-1 cells. **Figure S2.** Cellular responses evoked by the NPs in A549 cells. **Figure S3.** Dose-dependent airway inflammation induced by NP6 in the mouse.

## Data Availability

All data generated or analysed during this study are included in this published article and its supplementary information file.

## Abbreviations

BALFs: Bronchoalveolar lavage fluids
bPEI: Branched polyethylenimine
CDCD: Carbon dot
CI: Confidence interval
CLSM: Confocal laser scanning microscopy
DLS: Dynamic light scattering
DMEDA: *N,N*-Dimethylethylene diamine
mPEG: Poly(ethylene glycol) monomethyl ether
FACS: Fluorescence activated cell sorting
Q_ek_: Surface charge density
MWCO: Molecular weight cut-off
NP: Nanoparticle
PAA: Poly(acrylic acid)
PEHA: Pentaethylenehexamine
PES: Polyethersulfone
PMA: Phorbol 12-myristate 13-acetate
TEM: Transmission electron microscopy
