# Supplementary material for: Density of surface charge is a more predictive factor of the toxicity of cationic carbon nanoparticles than zeta potential
Source: J Nanobiotechnology. 2021 Jan 6;19:5. doi: 10.1186/s12951-020-00747-7 (PMC7789233; doi:10.1186/s12951-020-00747-7)
Supplement: Supplementary file 1 — Additional file: Figure S1. Cell uptake of NPs by A549 cells. Table S1. Cytokine production evoked by the NPs in THP-1 cells. Figure S2. Cellular responses evoked by the NPs in A549 cells. Figure S3. Dose-dependent airway inflammation induced by NP6 in the mouse. [file 12951_2020_747_MOESM1_ESM.pdf]

Additional file

Density of surface charge is a more predictive factor of the toxicity of cationic nanoparticles than zeta potential

Maud Weiss[a], Jiahui Fan[a], Mickaël Claudel[a], Thomas Sonntag[a], Pascal Didier[b], Carole Ronzani[a], Luc Lebeau[a], Françoise Pons[a,*]

[a]Laboratoire de Conception et Application de Molécules Bioactives, UMR 7199, CNRS-Université de Strasbourg, Faculté de Pharmacie, Illkirch, France

[b]Laboratoire de Bioimagerie et Pathologies, UMR 7021, CNRS-Université de Strasbourg, Faculté de Pharmacie, Illkirch, France

* Corresponding author

Full postal address: UMR 7199, Faculté de Pharmacie, 74 route du Rhin, 67400 Illkirch, France

E-mail: pons@unistra.fr

Phone: (+33) 3 68 85 42 03

Fax : (+33) 3 68 85 43 06

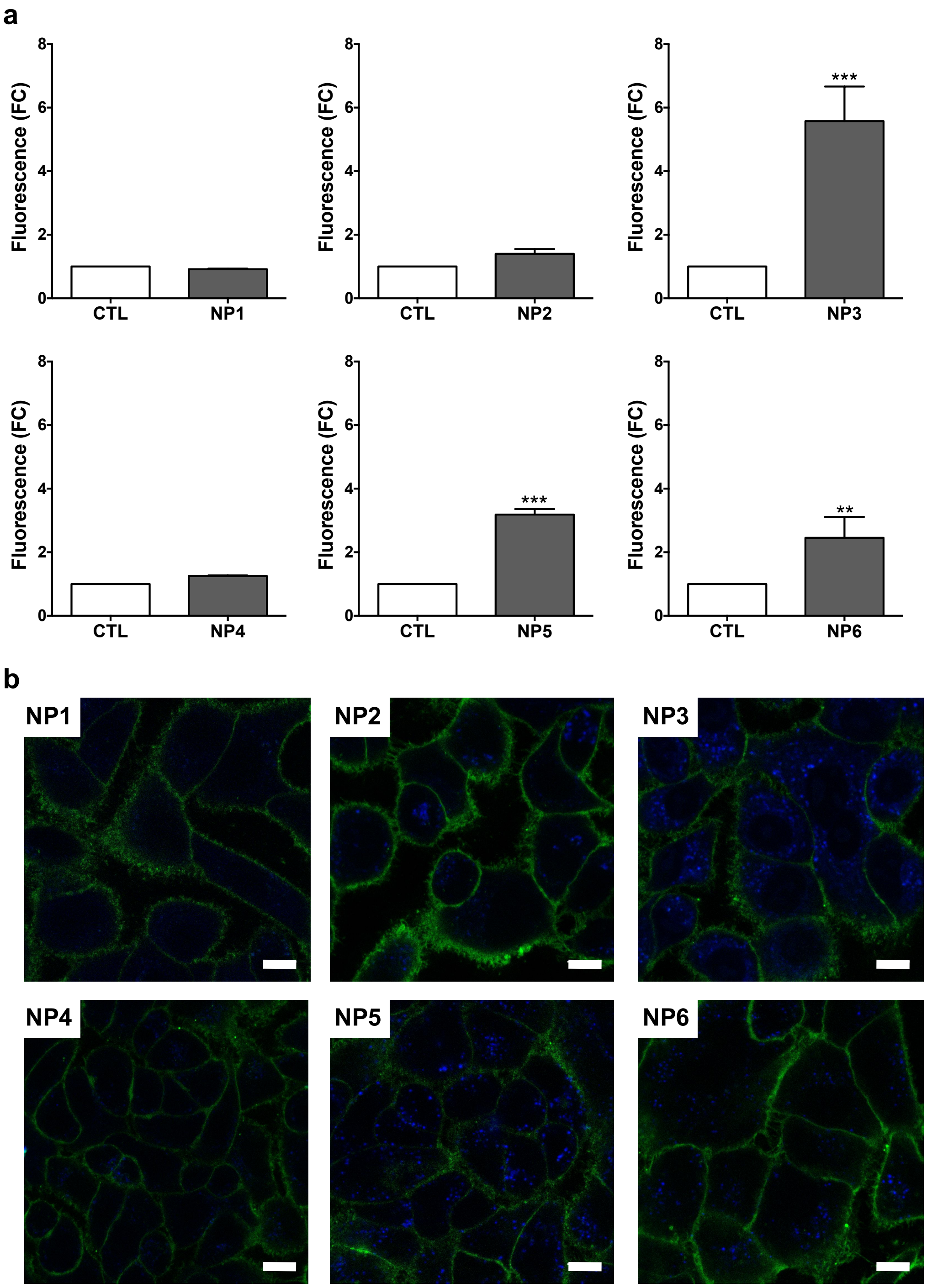

Figure S1. Cell uptake of NPs by A549 cells. **a** quantification of NP internalization by FACS. Results are expressed as fold change in fluorescence intensity when compared to control cells (CTL). They are means ± SEM of n = 3 experiments. Statistical differences when compared to control were determined by one-way ANOVA followed by the Dunnett's test. ** $p < 0.01$; *** $p < 0.001$. **b** CLSM of cells exposed to NPs. The cell membrane is colored in green thanks to the fluorescent probe DSQ12S and NPs appear in blue. All scale bars: 10 µm.

Table 1S. Cytokine production evoked by the NPs in THP-1 cells. Cells were exposed to 200 µg/mL NPs for 24 h and cytokines were assessed using a multi-analyte ELISArray. Samples were pools of n = 6 replicates and were tested once.

	N[illegible]	NP2	NP3	NP5	NP6
TNF-α	nd	nd	nd	nd	nd
IL-6	nd	nd	nd	nd	nd
IL-12	nd	nd	nd	nd	nd
IL-17	nd	nd	nd	nd	nd
Eotaxin	nd	nd	nd	nd	nd
MDC	-	-	-	-	-
IL-1β	-	-	-	+++	++
MIP-1α	-	-	+	+++	++
MIP-1β	-	+	+	+++	++
MCP-1	-	-	+++	++	+++

nd: non detectable. (-) : no change. (+): 1.5 < OD < 5-fold of the negative control or 0.10 < OD < 0.25-fold of the positive control; (++): 5 < OD < 10-fold of the negative control or 0.25 < OD < 0.75-fold of the positive control. (+++): OD > 10-fold of the negative control or OD > 0.75-fold of the positive control.

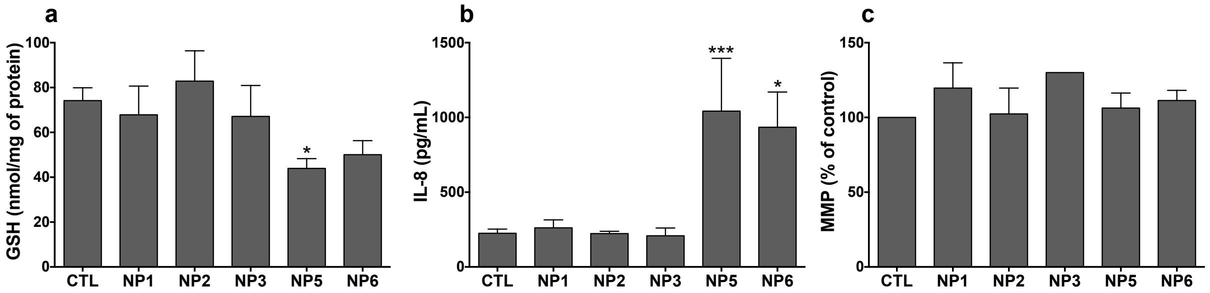

Figure S2. Cellular responses evoked by the NPs in A549 cells. Cells were exposed or not (CTL) to the NPs (100 µg/mL) for 4 h (**a** and **c**) or 24 h (**b**) and oxidative stress (**a**, reduced glutathione), inflammation (**b**, IL-8 secretion), and mitochondrial perturbation (**c**, mitochondrial membrane perturbation (MMP)) were measured as described in the method section. Data are means ± SEM of n = 3-6 experiments. They are expressed as absolute values (**a** and **b**) or in percentage of controls (**c**) . Statistical differences when compared to controls were determined by one-way ANOVA followed by the Dunnett's test. * $p < 0.05$; *** $p < 0.001$.

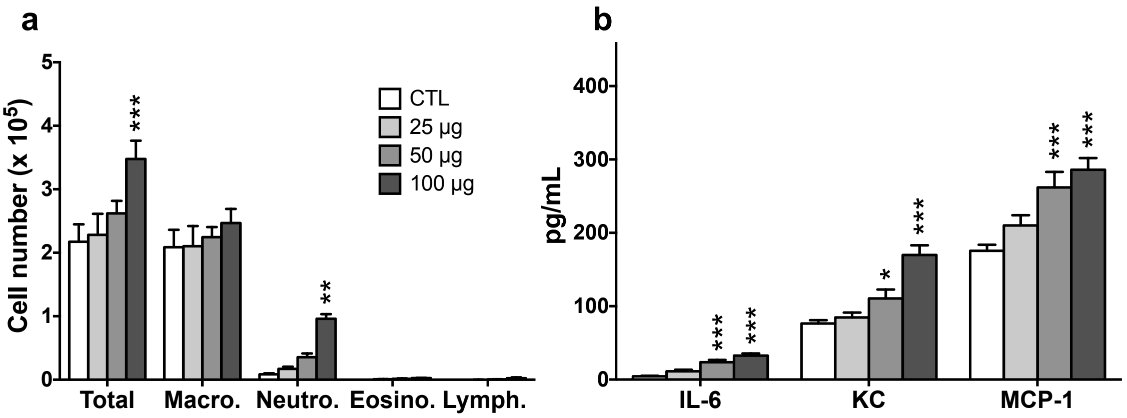

Figure S3. Dose-dependent airway inflammation induced by NP6 in the mouse. Mice received a single lung administration of of NP6 (25, 50 or 100 µg) or saline (CTL), and airway inflammation was assessed 24 h later by counting cells (**a**) and measuring cytokines (**b**) in bronchoalveolar lavage fluids. Data are means ± SEM of n = 6 mice. Statistical differences when compared to controls were determined by one-way ANOVA followed by the Dunnett's test. * $p < 0.05$; ** $p < 0.01$; *** $p < 0.001$.
